# Accelerating Decreases in the Incidences of Hepatocellular Carcinoma at a Younger Age in Shanghai Are Associated With Hepatitis B Virus Vaccination

**DOI:** 10.3389/fonc.2022.855945

**Published:** 2022-04-04

**Authors:** Shunzhang Yu, Qirong Zhu, Ying Zheng, Chunxiao Wu, Hong Ren, Xing Liu, Zhenqiu Liu, Yanting Li, Qichao Pan, Ying-Jie Zheng

**Affiliations:** ^1^ School of Public Health, Fudan University, Shanghai, China; ^2^ Children’s Hospital, Fudan University, Shanghai, China; ^3^ Department of Cancer Prevention, Fudan University Shanghai Cancer Center, Shanghai, China; ^4^ Cancer Prevention and Control, Shanghai Municipal Center for Disease Control and Prevention, Shanghai, China

**Keywords:** hepatitis B virus, mother–infant transmission, hepatitis B vaccination, hepatocellular carcinoma (HCC), prevention

## Abstract

**Background:**

Routine vaccination of infants for protecting against hepatitis B virus (HBV) infection and its serious consequences, including hepatocellular cancer (HCC), has been carried out in Shanghai, China, since 1986. We therefore have examined the trend of HBV infection and HCC incidences before and after HBV vaccination over decades to assess the potential influences of the Shanghai HBV vaccination program.

**Methods:**

Data on incidences of HBV infection and HCC were collected from the Shanghai Cancer Registry and the Shanghai HBV vaccination follow-up study. Joint-point regression and the Bayesian age-period-cohort statistical analysis methods were used.

**Results:**

The incidences of HBV infection dramatically declined from 23.09 and 1.13 per 100,000 for males and females in 2000 to 3.24 (-85.97%) and 0.22 (-80.53%) per 100,000 in 2014, respectively. Sero-epidemiological data from the sampling surveys during 20 years of follow-up showed that less than 1% of people undergoing HBV vaccination have a positive serum HBsAg. Consistently, the annual adjusted standardization rates (ASR) of HCC steadily fell from 33.38 and 11.65 per 100,000 for males and females in 1973 to 17.34 (-49.2%) and 5.60 (-51.9%) per 100,000 in 2014, respectively. The annual percentage change in overall HCC incidences is about -2%. HCC incidences in males at younger age groups (age <50 years old), particularly in those with age <34 groups, showed an accelerating decrease over time, whereas HCC incidences significantly declined in the female population across all age groups except for those under 19 years of age. The results supported that the universal HBV vaccination in newborns is easy to implement with high coverages and is effective for preventing both HBV infection and HCC in populations.

## Introduction

Primary liver cancer including hepatocellular carcinoma (HCC, 75%–85%) and intrahepatic cholangiocarcinoma (10%–15%), as well as other rare types, is the sixth most diagnosed malignancy and the third most common cause of cancer death in the world (1). According to the International Agency for Research on Cancer (IARC) report, approximately 906,000 new cases of primary liver cancer were diagnosed worldwide in 2020, about half of them in China (1). HCC is a fatal disease; less than 5% of HCC cases survive over a year after their initial diagnosis (2). The mortality and incidence of HCC are very similar, presenting with an annual fatality ratio of 0.97 (2). Therefore, primary prevention of HCC is a clear priority to effectively reduce the disease burden.

Up to 80% of all cases of HCC worldwide have HBV and/or HCV infection. Adults who have had a chronic HBV infection since childhood develop HCC at a rate of 5% per decade, which is 100–300 times the rate among uninfected people (3, 4). Therefore, HBV is one of the most known human carcinogens only second to tobacco smoking (3, 4). Because HBV most commonly spreads from mothers to infants or to those in their early childhood and the substantial burden of HBV-related diseases developed from HBV infections in childhood, universal vaccination in infants and children has been a most effective way to prevent HBV infection and reduce the prevalence of HBsAg and incidence of HCC (5).

The first HBV vaccine was blood-derived and approved in the United States in 1981 (6). Due to the concern about the possibility of introducing viral DNA into the vaccine, the safer recombinant HBV vaccine that contains the non-infectious surface protein of HBV was then generated in the yeast *Saccharomyces cerevisiae* to replace the blood-derived HBV vaccine in 1986 in the United States (7). In 1991, the Advisory Committee on Immunization Practices (ACIP) in the United States recommended universal childhood vaccination for eliminating HBV transmission in the United States (8). Now, many countries have implemented a routine HBV vaccination program in infants against chronic HBV infection and HCC (5, 9–11). Notably in Taiwan, China, with high rates of HBV infection, a universal HBV vaccination program has been launched in the region since 1984, which resulted in a significant reduction of HBV infection and the incidence of childhood HCC (5). Shanghai is one of the first cities in mainland China that started the hepatitis B immunization program in infants born to HBsAg-positive mothers in 1986 (12). A universal infant immunization program then extended to the whole Shanghai city in 1989 (13). Here we analyzed the impact of hepatitis B immunization in infants on the incidences of HBV infection and HCC after three decades of the vaccination in Shanghai. Our results show that the incidence of HBV infection in the Shanghai population decreased by more than 80% and age-standardized rates (ASRs) of HCC by around 50%. There is also an accelerating decrease in the incidence of HCC at the young male population in Shanghai.

## Methods

### Sources of Data

The Shanghai Cancer Registry was established by the Shanghai Tumor Hospital in the early 1960s and became a population-based registry in 1972. Since 1973, the incident cases of cancers and their mortality data have covered approximately all the permanent residents in Shanghai city. In 2002, a computer-based data collection system was established and used for the Shanghai Cancer Registry by the Shanghai Municipal Center for Disease Control and Prevention (CDC). The quality of submitted data for each local registry was examined by the registration officers at the Shanghai Municipal CDC according to the published guidelines by the International Agency for Research on Cancer (IARC) and the Chinese National Cancer Registry Center (14, 15). The ICD-9 coding system was used before 2002. Between 2002 and 2008, ICD-10 and ICD-O-2 were used. ICD-O-3 has been implemented since 2008. Cancer diagnosis data are received by regional CDCs from local hospitals and community centers. Mortality data are based on death certificates, which include information regarding primary cancer site, metastasis, follow-up, and other cancer-related clinical, pathological, and treatment information.

The quality of cancer report in each year from 1973 to 2013 in Shanghai was evaluated by 1) death certificate only, DCO%, 2) histological verification, HV%, and 3) the mortality-to-incidence ratio, M/I%, according to the published method by IARC (15). The data were presented as shown in **Supplementary Table 1** and **Supplementary Figure 1**.

### Shanghai Hepatitis B Vaccination Program

Three different types of hepatitis B vaccines have been used since 1986 in Shanghai. The first hepatitis B vaccine was derived from the plasma of patients with chronical HBV infection and produced by the Shanghai Biological Manufacture. The second one, named as the Recombivax hepatitis B vaccine, was a purified hepatitis B virus surface antigen (HBsAg) that was expressed by *Saccharomyces cerevisiae* (i.e., common baker’s yeast). The third one is a heat-inactivated and whole recombinant *Hansenula polymorpha* yeast expressing HBsAg.

The plasma-derived Hepatitis B vaccine was first tested in the field in 7,470 infants born in 1986 at Huangpu district (formally named as the Nanshi district), Shanghai. These hepatitis B-vaccinated infants were then followed up for the changes of HBV immunity, including HBsAg and anti-HBs. In addition, a cohort of infants born at a neighboring district Changning, Shanghai, without hepatitis B vaccination in the same year was used as the baseline control group in this study. In 1989, the plasma-derived hepatitis B vaccine inoculation was expanded to infants from 12 districts and counties in Shanghai. In 1995, a uniformed vaccine schedule and record with the first dose to be administered within 24 h of birth and subsequent doses at ages of 1 and 6 month(s) were developed for all infants in Shanghai city and a total of 128,000 subjects were inoculated with the plasma-derived hepatitis B vaccine with a full-course inoculation rate of over 99%.

In 1997, a yeast-derived recombinant hepatitis B vaccine was imported into China to replace the plasma-derived hepatitis B vaccine and a total of 81, 661 neonates were inoculated within 24 h of birth. In 2001, a total of 158,239 subjects, including neonates, children at ages of 13 and 17, and university freshmen in Shanghai, were inoculated with the hepatitis B vaccine. Since 2002, the hepatitis B vaccination program has become a part of the National Immunization Program of China. Since then, around 200,000 or more neonates each year in Shanghai have been inoculated with recombinant hepatitis B vaccine with a full-course inoculation rate of 99% or more.

### Hepatitis B Vaccination Follow-Up Study in Huangpu and Changning Districts, Shanghai

Blood samples had been collected from the hepatitis B-vaccinated infants at Huangpu district, Shanghai, and the hepatitis B-unvaccinated infants (baseline control group) at Changning district once every 2 years for a total of 28 years through a sampling approach. Serum HBsAg, anti-HBs, and anti-HBc levels were determined using enzyme-linked immunosorbent assay (ELISA) kits from Abbott Laboratories (Abbott Park, IL, USA), according to the manufacturer’s protocol. The anti-HBs cutoff value was ≥10.0 mIU/ml, and the anti-HBc sample value/cutoff value (S/CO) was ≥1.00.

### Statistical Analysis

The incidence of HCC among the population in each birth cohort was calculated by dividing the number of HCC cases by the total number of person years observed when the subjects were including the total population within Shanghai city. The standardized incidence rates in different diagnostic years ranging from 1973 to 2012 were adjusted by the 1960 world standard population. The trend of HCC incidences before (1973 to 2006) and after (1973 to 2012) hepatitis B vaccination was analyzed by joint-point regression analysis, as described by Kim et al. (16).

The Bayesian Age-Period-Cohort approach was used to analyze changes in HCC incidences in Shanghai by gender, age group, time period, and birth cohort and to project the incidence and mortality rates of HCC in Shanghai from years 2013 to 2020 based on the demographic shifts. The Poisson linear model was applied to estimate the annual percentage change (EAPC). These analyses were conducted with EAPC (17, 18) in Epi software package and INLA (19, 20) procedure in R software package (Version 3.3.3).

Age-Period-Cohort model for incidences of HCC


$$\begin{array}{l} \log\;\lambda_{ijk}=\mu +a_{i}+p_{j}+c_{k}+\zeta_{ijk}\\ \lambda_{ijk}:i\;denote\;age\;group,\;j\;period,\;k\;birth\;cohort\\ Pearson:\;\chi^{2}=\sum_{i,j}\frac{{\left(O_{ij}-E_{ij}\right)}^{2}}{E_{ij}},\\ Deviance:G^{2}=\sum_{i,j}O_{ij}\ln\left(\frac{O_{ij}}{E_{ij}}\right) \end{array}$$

## Results

### The Trend of HCC and Different Types of Viral Hepatitis Incidences From 1973 to 2014 in Shanghai, China

**Figure 1:**
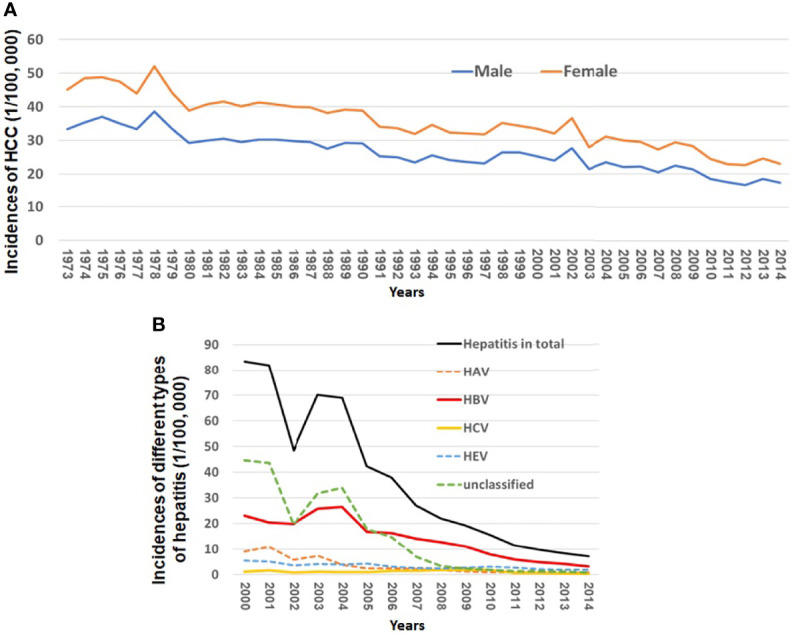
Incidences of HCC and different types of viral hepatitis from 1973 to 2014 in Shanghai City, China. **(A)** Incidences of HCC from 1973 to 2014 in Shanghai City and **(B)** incidences of HAV, HBV, HCV, HEV, and unclassified hepatitis from 2000 to 2014 in Shanghai City.

**Figure 1A** and **Supplementary Table 2** show a continuously decreasing trend of HCC incidences from 1973 to 2014 in Shanghai City, China. The age-standardized rates (ASRs) of HCC were 33.38 in males and 11.65 in females per 100,000 in 1973 compared to 17.34 in males and 5.60 in females in 2014, respectively. The incidences of male and female HCC decreased by 49.2% and 51.9% from 1973 to 2014, respectively.

Virus infection-related hepatitis can be divided into at least five types, namely, hepatitis A, B, C, and E and unclassified hepatitis. The incident cases of the viral infection related to hepatitis in Shanghai have been reported to the Shanghai Municipal CDC since 2000. The incidences of such viral infection in total decreased by 91.48% from 83.41 per 100,000 in 2000 to 7.11 per 100,000 in 2014 (**Figure 1B**). Among all types of hepatitis, the chronic infection of HBV or HCV plays a major role in development of HCC. **Figure 1B** and **Supplementary Table 3** show that the incidences of new HBV and HCV infection declined by 85.96% from 23.0 per 100,000 in 2000 to 3.24 in 2014 per 100,000 and by 80.53% from 1.13 per 100,000 to 0.22 per 100,000 in 2014, respectively.

### The Protective Effect of HBV Vaccine Against HBV Infection in the HBV-Vaccinated 1986 Birth Cohort in Huangpu District Compared to the Unvaccinated 1984 Baseline Birth Cohort in Changning District, Shanghai

The vaccine-induced immunity against HBV infection in the population was evaluated by three serum biomarkers, namely, HBsAg and anti-HBs and anti-HBc antibodies, from subjects who were selected randomly or non-randomly from the name lists of HBV-vaccinated infants (based on the consents or availability of the study subjects) in the population and followed up for 20 years since their HBV vaccination. **Table 1** shows that the positive rates of HBsAg were less than 1% ranging from 0.19% to 0.98% and that the positive rates of anti-HBs antibody and anti-HBc antibody were from 20. 65% to 89. 01%, and from 0. 96% to 3. 40%, respectively, during the past 20 years after the HBV vaccination in the population.

**Table 1:** Positive rates of HBV immunity-related biomarkers in randomly selected groups each year in the follow-up cohort of 7470 HBV-vaccinated infants in Shanghai (12).

Years after vaccination	HBsAg		Anti-HBs			Anti-HBc	
	No. of person follow-up	(+) %	No. of person follow-up	(+) %	GMT (mIU/mL)	No. of person follow-up	(+) %
1	432	0.45	282	89.01	148	–	–
3	716	0.98	420	78.57	62.4	–	–
5	988	0.51	716	59.92	31.8	628	3.40
7	413	0.97	408	47.06	33.3	411	1.95
8	213	0.94	213	41.31	14.6	282	1.42
9	374	0.53	374	40.37	13.4	374	2.67
11	442	0.68	367	45.58	16.9	441	1.13
13	5320	0.19	–	–	–	520	0.96
14	410	0.73	410	29.02	–	410	1.95
16	422	0.47	422	27.25	–	422	1.18
18	402	0.50	402	30.10	–	402	1.24
20	368	0.54	368	20.65	–	368	1.90
Average	5,700	0.61	4,976	41.66	45.77	4 258	1.83

**Figure 2:**
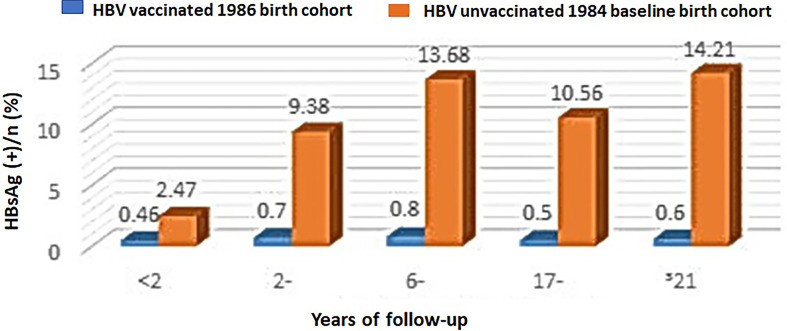
HBsAg-positive rates in the HBV-vaccinated 1986 birth cohort at Huangpu district *versus* the unvaccinated 1984 birth cohort at Changning district in Shanghai City, China.

In addition, infants born in a neighboring district Changning, Shanghai City, in 1984 served as a baseline control birth cohort. **Figure 2** shows that the HBsAg-positive rates in different ages in the 1986 birth cohort at Huangpu district were all below 0.8%, whereas the HBsAg-positive rates in the unvaccinated population in Changning district exhibited an age-dependent increase from 2.47% for those age less than 2 years old to 14.21% for those age equal or more than 21 years old. This result supports a significant protective effect of HBV vaccination against HBV infection in the 1986 birth cohort at Huangpu district.

### The Decreasing Rates of HCC Incidences Before and After HBV Vaccination and in Different Age Groups in Shanghai

Universal hepatitis B vaccination was extended to all newborns and school students under the age of 12 years in Shanghai to protect against HBV infection and its complications including liver cirrhosis and HCC. We therefore analyzed rates of change in HCC incidences before and after the universal HBV vaccination in Shanghai City by using joint-point regression analysis. Regression coefficient β represents the rate of changes in HCC incidences during the indicated periods shown in **Table 2**. There is no significant difference in the overall decreasing rates of HCC incidences in all age groups between periods of 1979 to 1999 and 2000 to 2012 (**Table 3**). However, HCC incidences in the age 0–14, 15–19, and 20–29 groups exhibited an accelerated decrease with the negative two digital regression coefficients. The regression coefficients in age between 30 and 49 and in age of 50 and more are negative for a single digital and less than a single digital, respectively. This result suggested that the HCC incidences dropped significantly in all age groups during the past years in Shanghai, while the same rates in those aged 29 and below had an accelerated decline.

**Table 2:** The trend of HCC incidences before and after HBV vaccination in Shanghai.

Duration	HBV vaccination	Regression coefficient	t-test	Sig.
		β ± S.E.		
1979–1999	Before	-1.901 ± 0.285	-6.680	.000
2000–2012	After	-1.090 ± 0.228	-4.772	.001

**Table 3:** The regression trends of HCC incidences in different age groups in Shanghai.

Age group	Coefficient (β ± S.E.)	p value
0~	-25.51 ± 9.49	0.043
15~	-24.27 ± 5.12	0.005
20~	-17.10 ± 2.04	0.000
30~	-5.76 ± 1.50	0.018
35~	-1.81 ± 0.67	0.043
40~	-1.00 ± 0.35	0.036
50~	-0.59 ± 0.18	0.021
55~	-0.52 ± 0.08	0.001
60~	-0.42 ± 0.02	0.000
65~	-0.46 ± 0.08	0.003
70~	-0.54 ± 0.14	0.012
75~	-0.84 ± 0.31	0.023
80~	0.43 ± 0.13	0.023
85~	0.20 ± 0.04	0.005

In addition, we have carried out a birth cohort analysis for changes of HCC incidences during the period of 1973–2012. **Tables 4A**, **B** show that the incidences of HCC in all age groups decreased over time and the estimated annual percentage changes in the male age group from 20 to 34 and the female age group from age more than 20, respectively, had the most significant decrease.

**Table 4A:** Age-standardized rates (ASR) of HCC in Shanghai from 1973 to 2012.

Sex	Period (year)	Cases	ASR	Crude rate (/10^5^)	ASR (/10^5^)					Total rates (35–64 years)
			(/10^5^)		0–19	20–34	35–49	50–64	65+	
Male										
	1973–1982	10,781	33.59	37.39	0.62	2.94	29.90	87.66	151.22	70.62
	1983–1992	12,337	28.54	35.31	0.40	2.87	20.23	72.88	150.99	55.03
	1993–2002	11,757	25.07	36.40	0.22	2.02	20.58	58.66	142.03	47.49
	2003–2012	12,469	20.65	40.28	0.33	1.11	15.99	50.12	118.93	39.53
	Total	47,344	26.89	36.98	0.39	2.12	19.46	66.79	143.39	52.49
Female										
	1973–1982	4,021	11.53	14.28	0.26	0.74	6.18	25.82	76.98	19.09
	1983–1992	4,926	10.03	14.69	0.23	0.82	4.18	22.16	72.46	15.46
	1993–2002	4,733	8.48	15.11	0.30	0.53	3.87	16.01	68.04	11.95
	2003–2012	5,012	6.63	16.23	0.20	0.40	2.79	11.38	58.97	8.45
	Total	18,692	8,89	15.02	0.25	0.61	3.99	18.32	68.89	15.17

**Table 4B:** The estimated annual percentage changes (EAPC) of liver cancer incidence in Shanghai from 1973 to 2012.

Sex	Period	EAPC (95% CI)				
		0–19	20–34	35–49	50–64	65+
Males						
	1973–1982	-5.87 (-21.95, 13.52)	-2.84 (-7.34, 1.87)	-4.60 (-7.74, -1.35)	-2.42 (-4.90, 0.12)	0.77 (-1.02, 2.60)
	1983–1992	9.10 (-8.66, 30.32)	-0.37 (-3.86, 3.24)	-3.31 (-6.30, -0.23)	-2.11 (-3.12, -1.10)	-0.65 (-3.03, 1.77)
	1993–2002	-19.65 (-35.88, 0.70)	-3.84 (-10.51, 3.33)	1.99 (-0.38, 4.43)	1.81 (-0.30, 3.97)	1.10 (-0.41, 2.64))
	2003–2012	3.78 (-8,83, 7.35)	-6.08 (-14.29, 2.91)	-6.48 (-9.21, -3.66)	-1.70 (-3.47, 0.10)	-5.22 (-6.38, -4.05)
	Total	-1.68 (-4.17, 0.88)	-3.39 (-4.16, -2.60)	-1.94 (-2.40, -1.47)	-1.82 (-2.07, -1.58)	-0.82 (-1.12, -0.52)
Females						
	1973–1982	-2.92 (-27.06, 29.21)	0.40 (-5.72, 6.93)	-4.09 (-8.57, 0.60)	-1.26 (-4.57, 2.17)	-1.89 (-4.68, 0.98)
	1983–1992	-2.46 (-22.32, 22.47)	-1.30 (-8.87, 6.90)	-2.74 (-7.60, 2.38)	-2.44 (-3.97, -0.89)	-1.51 (-2.72, -0.28)
	1993–2002	15.43 (-13.28, 53.67)	5.18 (-6.10, 17.83)	1.66 (-3.91, 7.55))	0.63 (-1.18, 2.48)	-0.80 (-2.36, 0.78)
	2003–2012	20.44 (-16.02, 72.73)	-2.59 (-15.70, 12.57)	-3.02 (-7.67, 1.86)	-3.80 (-7.53, 0.07)	-4.38 (-6.47, -2.24)
	Total	-0.51 (-3.39, 2.47)	-2.37 (-3.50, -1.23)	-2.40 (-2.96, -1.83)	-2.71 (-3.06, -2.35)	-0.95 (-1.22, -0.69)

## Discussion and Conclusion

Chronic HBV infection has been a major public health issue in China. The majority is infected *via* mother-to-child transmission in endemic areas (21). The risk of developing chronic infection was also inversely related to the age onset as approximately 90% of individuals who are infected perinatally with HBV are likely to develop chronic infection (21). Therefore, a key strategy for the prevention of HBV chronic infection is universal HBV vaccination in infants beginning at birth (5). To protect people from HBV infection, a plasma-derived inactivated hepatitis B vaccine was first produced in Shanghai, China, in 1983 and 7,470 newborns in Huangpu district, Shanghai City, were inculcated with the vaccine within 24 h and at 1 and 6 month(s) after birth in 1986. The coverage rates of the three-dose hepatitis B vaccine reached to 95% or more. HBsAg-positive rates in the population ranged from 0.19% to 0.98% during the 20 years of follow-up after the vaccination, whereas the HBsAg-positive rates in the unvaccinated population at Changning district, Shanghai, were at higher levels ranging from 2.47% to 14.21%. The HBV vaccination-related seroprevalence of anti-HBs ranged from 89.01% to 20.65% positive rates during the 20 years of follow-up. In some subjects, the protective anti-HBs lasted for at least 20 years and the antibody titrations were more than 10 mIU/ml in the recipients’ serum. Our results clearly indicate that the plasma-derived inactivated hepatitis B vaccine has provided highly protective effects for newborns against HBV infection.

Chronic HBV infection is a major risk factor for HCC. The hepatitis B virus integrates its viral DNA into the host genome and induces chronic necroinflammation and mutations during the infection, which can progress to HCC over a period of decades (22). The lifetime risk of chronic HBV infection for developing HCC was estimated to be between 10% and 25% (4). Therefore, universal hepatitis B vaccination in newborns is an obvious key strategy for primary prevention of HCC. Indeed, Chiang et al. (23) reported a decrease in HCC incidence and mortality by 80% and 92%, respectively, 30 years after the initiation of the whole-Taiwan Hepatitis B Immunization Program in 1984. The Shanghai Hepatitis B Immunization Program started in 1986 with the plasma-derived inactivated hepatitis B vaccine given to newborns in Huangpu district. Coverage was then extended to all infants and preschool and primary schoolchildren using the uniformed vaccine schedule and records during 1989 to 1995. The plasma-derived inactivated hepatitis B vaccine was substituted by recombinant HBV vaccines in 1996, and the vaccine inoculation coverage reached to 96% of all three dosages in infants. Students at ages of 13 and 17 and university freshmen in Shanghai were also inoculated with the HBV vaccine in 1997 with 86% of the population covered. In 2001, more than 6 million infants in each year were inoculated with the recombinant HBV vaccine. The initiation of the Shanghai Hepatitis B Immunization Program was accompanied by a decrease in HCC incidences after 30 years by 49.2% in males and 51.9% in females, respectively. However, the reduction of HCC incidences in Shanghai (by about 50%) is less effective compared to the Taiwan Hepatitis B Immunization Program that is up to an 80% decrease in HCC incidence. The prevalence of HBsAg positivity is similar in the examined populations of these two HBV vaccination programs and less than 1%. This discrepancy suggests that other potential factors, such as food contamination with aflatoxin and algal hepatotoxins in drinking water, may still play an oncogenic role in HCC in Shanghai.

In addition, we observed an accelerated decrease in HCC incidences in young adults, in particular for groups of males with an age <34 30 years after the Shanghai Hepatitis B Immunization Program started. This observation is consistent with the reports from Taiwan and many other countries, such as Singapore and Spain, in their implemented HBV vaccination programs in 1980s (24–31). However, our results also showed a similar decrease in HCC incidences in the female population across all the age groups that are more than 19 years old. The results suggest that the HBV vaccination program may have different effects on the reduction in HCC incidence rates between males and females at different ages. Currently, there is very little information or studies for explaining this observation. It has been shown that the incidence rates of female HCC peak consistently by 5 years compared to those of male HCC across different areas of the world regardless of the large variation in their age distributions in different countries or regions (32). It is possible that the other risk factors such as alcohol consumption and tobacco smoking are less common in females and there is a significant difference in sex hormones and hormonal immunity. Such factors may play a role in the differential effects of HBV vaccination on males and females. Further studies are needed to confirm and investigate this phenomenon.

In conclusion, the universal HBV vaccination in newborns is easy to implement with high coverages and is very cost-effective for the prevention of both HBV infection and HCC in populations. However, in HCC-endemic areas, such as Shanghai, more comprehensive prevention measures like improvement of water quality and elimination of aflatoxin-contaminated foods, and drinking less alcohol are also needed to achieve better control of HCC incidences. In addition, the potential difference of HBV vaccination effects on male and female populations is very intriguing and deserves further investigation.

## Data Availability Statement

The raw data supporting the conclusions of this article will be made available by the authors, without undue reservation.

## Author Contributions

SY: project design, concept, analysis, funding resource, and manuscript writing. QZ: data collection and resources. YZ: data collection, analysis, and resources. CW: data collection and resources. HR: data collection and resources. XL: data collection and resource. ZL: data collection and resources. YL: data collection and resources. QP: data collection and resources. Y-JZ: data collection and resources. All authors contributed to the article and approved the submitted version.

## Conflict of Interest

WS is a member of the CT Advisory Board of Philips Medical Systems.

The remaining authors declare that the research was conducted in the absence of any commercial or financial relationships that could be construed as a potential conflict of interest.

## Publisher’s Note

All claims expressed in this article are solely those of the authors and do not necessarily represent those of their affiliated organizations, or those of the publisher, the editors and the reviewers. Any product that may be evaluated in this article, or claim that may be made by its manufacturer, is not guaranteed or endorsed by the publisher.
