# Corrigendum: Accelerating Decreases in the Incidences of Hepatocellular Carcinoma at a Younger Age in Shanghai Are Associated With Hepatitis B Virus Vaccination

**DOI:** 10.3389/fonc.2022.950499

**Published:** 2022-09-20

**Authors:** Shunzhang Yu, Qirong Zhu, Ying Zheng, Chunxiao Wu, Hong Ren, Xing Liu, Zhenqiu Liu, Yanting Li, Qichao Pan, Ying-Jie Zheng

**Affiliations:** ^1^ School of Public Health, Fudan University, Shanghai, China; ^2^ Children’s Hospital, Fudan University, Shanghai, China; ^3^ Department of Cancer Prevention, Fudan University Shanghai Cancer Center, Shanghai, China; ^4^ Cancer Prevention and Control, Shanghai Municipal Center for Disease Control and Prevention, Shanghai, China

**Keywords:** hepatitis B virus, mother–infant transmission, hepatitis B vaccination, hepatocellular carcinoma (HCC), prevention

In the published article, there was an error in author Ying Zheng's affiliation. Instead of “Cancer Prevention and Control, Shanghai Municipal Center for Disease Control and Prevention, Shanghai, China”, it should be “Department of Cancer Prevention, Fudan University Shanghai Cancer Center, Shanghai, China”.

In the published article Shunzhang Yu and Xiaolin Zi were listed as corresponding authors; however, the only corresponding author is Shunzhang Yu and Xiaolin Zi was removed from the author list.

The authors apologize for these errors and state that this does not change the scientific conclusions of the article in any way. The original article has been updated.

## Publisher’s Note

All claims expressed in this article are solely those of the authors and do not necessarily represent those of their affiliated organizations, or those of the publisher, the editors and the reviewers. Any product that may be evaluated in this article, or claim that may be made by its manufacturer, is not guaranteed or endorsed by the publisher.

