# Supplementary material for: Accelerating Decreases in the Incidences of Hepatocellular Carcinoma at a Younger Age in Shanghai Are Associated With Hepatitis B Virus Vaccination
Source: Front Oncol. 2022 Apr 4;12:855945. doi: 10.3389/fonc.2022.855945 (PMC9014260; doi:10.3389/fonc.2022.855945)
Supplement: Supplementary file 1 [file DataSheet_1.docx]

**Supplementary Table 1**, The quality control of HCC cancer report in Shanghai

| Year Diagnosis | DCO | | D/C | | HV | |
| --- | --- | --- | --- | --- | --- | --- |
|  | N | ％ | N | ％ | N | ％ |
| 1973 | 386 | 29.58 | 1305/1282 | 0.97 | 179 | 13.72 |
| 1974 | 571 | 38.32 | 1490/1391 | 0.93 | 139 | 9.33 |
| 1975 | 649 | 42.61 | 1523/1500 | 0.98 | 88 | 5.78 |
| 1976 | 688 | 46.11 | 1492/1492 | 0.99 | 181 | 12.13 |
| 1977 | 649 | 46.09 | 1408/1405 | 0.99 | 140 | 9.94 |
| 1978 | 740 | 43.81 | 1689/1688 | 1.01 | 167 | 9.89 |
| 1979 | 503 | 33.56 | 1499/1595 | 1.04 | 146 | 9.74 |
| 1980 | 578 | 41.67 | 1387/1526 | 1.08 | 134 | 9.66 |
| 1981 | 627 | 42.97 | 1459/1463 | 1.00 | 117 | 8.02 |
| 1982 | 717 | 46.26 | 1550/1489 | 0.97 | 117 | 7.55 |
| 1983 | 730 | 47.22 | 1546/1578 | 1.02 | 125 | 8.09 |
| 1984 | 725 | 44.78 | 1619/1589 | 0.96 | 130 | 8.03 |
| 1985 | 800 | 46.14 | 1734/1675 | 0.95 | 133 | 7.67 |
| 1986 | 840 | 47.57 | 1766/1713 | 0.96 | 145 | 8.21 |
| 1987 | 890 | 49.67 | 1792/1804 | 1.00 | 150 | 8.37 |
| 1988 | 204 | 11.54 | 1767/1666 | 0.93 | 344 | 19.47 |
| 1989 | 72 | 4.14 | 1740/1739 | 0.97 | 199 | 11.44 |
| 1990 | 77 | 4.20 | 1834/1746 | 0.92 | 239 | 13.04 |
| 1991 | 14 | 0.82 | 1703/1794 | 1.07 | 225 | 13.21 |
| 1992 | 1 | 0.06 | 1762/1832 | 1.00 | 262 | 14.87 |
| 1993 | 0 | 0.00 | 1613/1657 | 1.00 | 213 | 13.21 |
| 1994 | 0 | 0.00 | 1677/1599 | 0.96 | 211 | 12.58 |
| 1995 | 0 | 0.00 | 1582/1568 | 1.00 | 191 | 12.09 |
| 1996 | 0 | 0.00 | 1606/1613 | 1.00 | 215 | 13.42 |
| 1997 | 0 | 0.00 | 1570/1518 | 0.95 | 211 | 13.44 |
| 1998 | 6 | 0.35 | 1710/1571 | 0.90 | 249 | 14.56 |
| 1999 | 0 | 0.00 | 1677/1519 | 0.89 | 252 | 15.03 |
| 2000 | 0 | 0.00 | 1631/1522 | 0.94 | 235 | 14.41 |
| 2001 | 1 | 0.06 | 1586/1521 | 0.93 | 283 | 17.84 |
| 2002 | 1 | 0.05 | 1838/1601 | 0.87 | 346 | 18.82 |
| 2003 | 1 | 0.06 | 1682/1466 | 0.85 | 344 | 20.45 |
| 2004 | 3 | 0.16 | 1832/1422 | 0.77 | 398 | 21.72 |
| 2005 | 2 | 0.11 | 1832/1417 | 0.78 | 394 | 21.51 |
| 2006 | 2 | 0.11 | 1804/1508 | 0.83 | 407 | 22.56 |
| 2007 | 2 | 0.11 | 1769/1537 | 0.84 | 425 | 24.02 |
| 2008 | 2 | 0.11 | 1821/1565 | 0.85 | 452 | 24.82 |
| 2009 | 0 | 0.00 | 1748/1566 | 0.91 | 469 | 26.83 |
| 2010 | 0 | 0.00 | 1757/1442 | 0.82 | 528 | 30.05 |
| 2011 | 1 | 0.06 | 1679/1458 | 0.85 | 491 | 29.24 |
| 2012 | 0 | 0.00 | 1557/1402 | 0.90 | 465 | 29.87 |
| 2013 | 138 | 0.08 | 1426/1705 | 0.84 | 642 | 37.65 |

**Supplementary Figure 1**


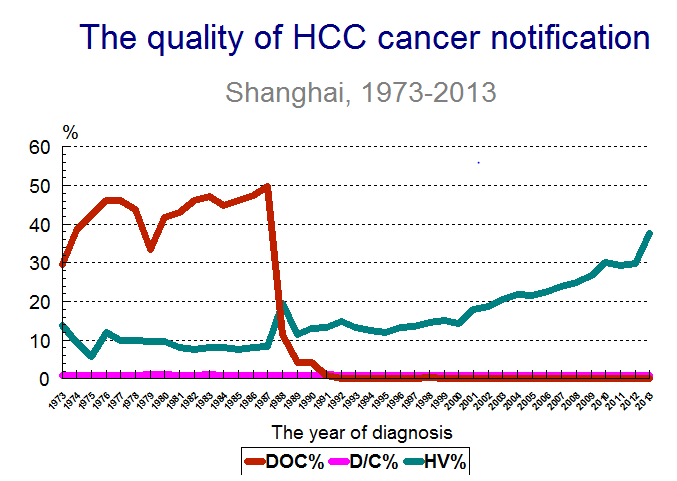


| **Year** | **HCC** | | | | | |
| --- | --- | --- | --- | --- | --- | --- |
|  | **Male** | | **Female** | | | |
| **1973** | **33.38** | | | **11.65** | |  |
| **1974** | **35.28** | | | **13.22** | |  |
| **1975** | **37.07** | | | **11.72** | |  |
| **1976** | **35.06** | | | **12.47** | |  |
| **1977** | **33.36** | | | **10.60** | |  |
| **1978** | **38.55** | | | **13.54** | |  |
| **1979** | **33.45** | | | **10.75** | |  |
| **1980** | **29.25** | | | **9.56** | |  |
| **1981** | **29.96** | | | **10.80** | |  |
| **1982** | **30.56** | | | **10.98** | |  |
| **1983** | **29.54** | | | **10.58** | |  |
| **1984** | **30.24** | | | **10.96** | |  |
| **1985** | **30.21** | | | **10.45** | |  |
| **1986** | **29.85** | | | **10.11** | |  |
| **1987** | **29.57** | | | **10.30** | |  |
| **1988** | **27.56** | | | **10.57** | |  |
| **1989** | **29.21** | | | **9.87** | |  |
| **1990** | **29.04** | | | **9.89** | |  |
| **1991** | **25.23** | | | **8.86** | |  |
| **1992** | **24.91** | | | **8.72** | |  |
| **1993** | **23.48** | | | **8.48** | |  |
| **1994** | **25.59** | | | **9.02** | |  |
| **1995** | **24.06** | | | **8.24** | |  |
| **1996** | **23.63** | | | **8.40** | |  |
| **1997** | **23.12** | | | **8.63** | |  |
| **1998** | **26.43** | | | **8.70** | |  |
| **1999** | **26.46** | | | | **7.93** | |
| **2000** | **25.26** | **8.25** | | | | |
| **2001** | **24.02** | **7.99** | | | | |
| **2002** | **27.62** | **8.91** | | | | |
| **2003** | **21.40** | **6.53** | | | | |
| **2004** | **23.47** | **7.61** | | | | |
| **2005** | **22.04** | **7.89** | | | | |
| **2006** | **22.18** | **7.29** | | | | |
| **2007** | **20.44** | **6.76** | | | | |
| **2008** | **22.44** | **6.89** | | | | |
| **2009** | **21.34** | **6.93** | | | | |
| **2010** | **18.51** | **5.93** | | | | |
| **2011** | **17.53** | | **5.27** | | | |
| **2012** | **16.63** | | **5.99** | | | |
| **2013** | **18.39** | | | | **6.14** | |
| **2014** | **17.34** | | | | **5.60** | |
|  |  | | | |  | |

**Supplementary Table 2**, incidences (ASRs, 1/100, 000) of HCC from 1973 to 2014 in Shanghai

**Supplementary Table 3**, incidences (1/100,000) of different types of hepatitis from 1973 to 2014 in Shanghai

| Year | **Hepatitis in total** | **HAV** | **HBV** | **HCV** | **HEV** | **unclassified** |
| --- | --- | --- | --- | --- | --- | --- |
| **2000** | 83.41 | 9.03 | 23.09 | 1.13 | 5.48 | 44.67 |
| **2001** | 81.83 | 10.95 | 20.33 | 1.65 | 5.21 | 43.68 |
| **2002** | 48.43 | 5.78 | 19.83 | 0.83 | 3.58 | 19.41 |
| **2003** | 70.09 | 7.29 | 25.73 | 1.21 | 4.11 | 31.76 |
| **2004** | 69.06 | 3.7 | 26.53 | 1.02 | 3.88 | 33.92 |
| **2005** | 42.37 | 2.45 | 16.85 | 0.99 | 4.24 | 17.84 |
| **2006** | 37.92 | 2.41 | 16.22 | 1.42 | 3.12 | 14.75 |
| **2007** | 26.99 | 2.1 | 14.03 | 1.42 | 2.54 | 6.91 |
| **2008** | 22.02 | 1.69 | 12.74 | 1.8 | 2.49 | 3.3 |
| **2009** | 19.21 | 1.31 | 11.08 | 1.81 | 2.71 | 2.3 |
| **2010** | 15.52 | 0.92 | 7.88 | 1.89 | 3.05 | 1.79 |
| **2011** | 11.39 | 0.83 | 5.93 | 0.63 | 2.71 | 1.29 |
| **2012** | 9.66 | 0.66 | 4.96 | 0.66 | 2.1 | 1.28 |
| **2013** | 8.39 | 0.55 | 4.2 | 0.51 | 1.9 | 1.23 |
| **2014** | 7.11 | 0.83 | 3.24 | 0.22 | 1.96 | 0.87 |
